# Nanospiral Formation by Droplet Drying: One Molecule at a Time

**DOI:** 10.1007/s11671-010-9793-9

**Published:** 2010-09-30

**Authors:** Lei Wan, Li Li, Guangzhao Mao

**Affiliations:** 1 Department of Chemical Engineering and Materials Science, Wayne State University, Detroit, Michigan 48202, USA

**Keywords:** Atomic-force microscopy, Interfaces, Nanostructures, Patterning, Templates, Self-assembly

## Abstract

We have created nanospirals by self-assembly during droplet evaporation. The nanospirals, 60–70 nm in diameter, formed when solvent mixtures of methanol and *m*-cresol were used. In contrast, spin coating using only methanol as the solvent produced epitaxial films of stripe nanopatterns and using only *m*-cresol disordered structure. Due to the disparity in vapor pressure between the two solvents, droplets of *m*-cresol solution remaining on the substrate serve as templates for the self-assembly of carboxylic acid molecules, which in turn allows the visualization of solution droplet evaporation one molecule at a time.

## Introduction

Patterns formed by solvent evaporation are relevant to various coating processes as well as patterning technology. In capturing the molecular process of an evaporating droplet, this work demonstrates the possibility to further modulate dewetting patterns by amphiphiles capable of self-assembly. Self-assembly as an alternative to lithography has the potential to generate reconfigurable nanostructures [1-3]. Surfactants/amphiphiles are the simplest molecules to self-assemble into complex yet often predictable structures and phases. An interface perturbs and sometimes dominates the self-assembling behavior of amphiphiles. A well-known example of substrate-dominated self-assembly is the epitaxial stripe nanopatterns formed by alkanes and alkane derivatives on highly oriented pyrolytic graphite (HOPG) [4-10]. The 1,3-methylene group distance, 0.251 nm, of all-trans alkyl chains matches the distance of the next nearest neighbor of the HOPG lattice, 0.246 nm, along, e.g., the [11$\bar{2}$0] crystallographic direction. The head-to-head arrangement gives rise to the stripe nanopattern whose periodicity is 1 × or 2 × the molecular chain length. Such nanopatterns serve as model templates for the study of site-specific adsorption, alignment, assembly, and reaction of small molecules [8,9,11,12] as well as macromolecules [13-16].

In an earlier example, we disrupted the stripe nanopattern of eicosanoic acid (C_20_A) using mercaptoundecanoic acid capped cadmium sulfide nanoparticles. C_20_A nanorods with 1.0 nm in thickness and 5.4 nm in width are nucleated directly on the nanoparticle to produce nanoparticle/nanorod hybrid structure [17]. Here, we present another method to perturb the epitaxial interaction between long-chain carboxylic acids and HOPG and to create spiral nanopatterns by adding a co-solvent to the spin coating solution. We propose that the curved nanostructure is formed at the receding solid/liquid/vapor contact line of an evaporating solution droplet, and it traces the entire droplet evaporation process at the molecular scale.

Recently, a number of methods have been reported for making circular nanostructures. Nanorings have been generated by lithography (microcontact printing [18], electron beam [19], and AFM tips [20]), template-based synthesis (using droplets [21], viruses [22], and DNA [23]), self-assembly [24-27], selective dewetting on patterned surfaces [28-30], and evaporation-driven dewetting [27,31-33]. There have been fewer reports on nanospirals [34-37]. The scientific interests for nanorings range from quantum rings, whose connected geometry at the nanoscale can trap "persistent currents" [38-41], to biomimetic light-harvesting complexes [31,42,43] and DNA microarrays for high-throughput DNA mapping [44,45]. The nanoring structure is also interesting because of its resemblance of the toroid structure of condensed DNA [26].

## Experimental Section

### Materials

Long-chain carboxylic acids including hexadecanoic acid (C_16_A, Aldrich, 99%), octadecanoic acid (C_18_A, Fluka, ≥ 99.5%), eicosanoic acid (C_20_A, Sigma, ≥99%), docosanoic acid (C_22_A, Aldrich, 99%), tetracosanoic acid (C_24_A, Fluka, ≥99.0%), and hexacosanoic acid (C_26_A, Sigma, ≥95%) were used. Solvents used were *m*-cresol (Aldrich, 97%), methanol (Mallinckrodt Chemicals, 100%), ethanol (Pharmco, 100%), *iso*-propanol (Fisher Scientific, 100%), and *sec*-butanol (Fisher Scientific, 99.3%). HOPG (grade ZYB) was purchased from MikroMasch. All chemicals were used as received.

### Sample Preparation

Carboxylic acids were dissolved in a primary alcoholic solvent or a binary solvent of alcohol and *m*-cresol to yield a final concentration of 0.2–0.4 mM. HOPG was freshly cleaved by adhesive tapes. The spin coating (PM101DT-R485 photoresist spinner, Headway Research) was conducted at room temperature in ambient air with relative humidity <40%. A volume of 100 μL of the solution was dispensed onto HOPG and spun at 3,000 rpm for 60 s. The samples were dried in air for 20 min or longer.

### AFM Characterization

The spin-coated samples were imaged using Nanoscope III Multimode AFM equipped with a piezoelectric scanner with a maximum scan range of 10 μm (*x* and *y*) and 2.5 μm (*z*) from VEECO/Digital Instruments. Height, amplitude, and phase images were obtained in Tapping Mode (oscillation frequency ~ 250–300 kHz) in ambient atmosphere using etched silicon probes (ACT, NanoScience) with nominal radius of curvature <10 nm. The scan rate was 1–3 Hz. Integral and proportional gains were approximately 0.4 and 0.8, respectively. Only flattened height images were shown. The films were usually imaged within minutes of film preparation. However, the nanostructures were unchanged for at least 1 month afterward when stored in ambient environment. The contour length of the stripe was determined using the WSxM 4.0 software.

### Contact Angle Measurement

The contact angle was measured by an NRL contact angle goniometer (Model 100, Rame-Hart) in the laboratory atmosphere. One *m*-cresol droplet of 5 μL was placed on the substrate and contact angles were read on both sides of the droplet. Five droplets were placed at various spots near the center of the substrate, and contact angles were averaged with an error of ±3°.

## Results and Discussion

**Figure 1:**
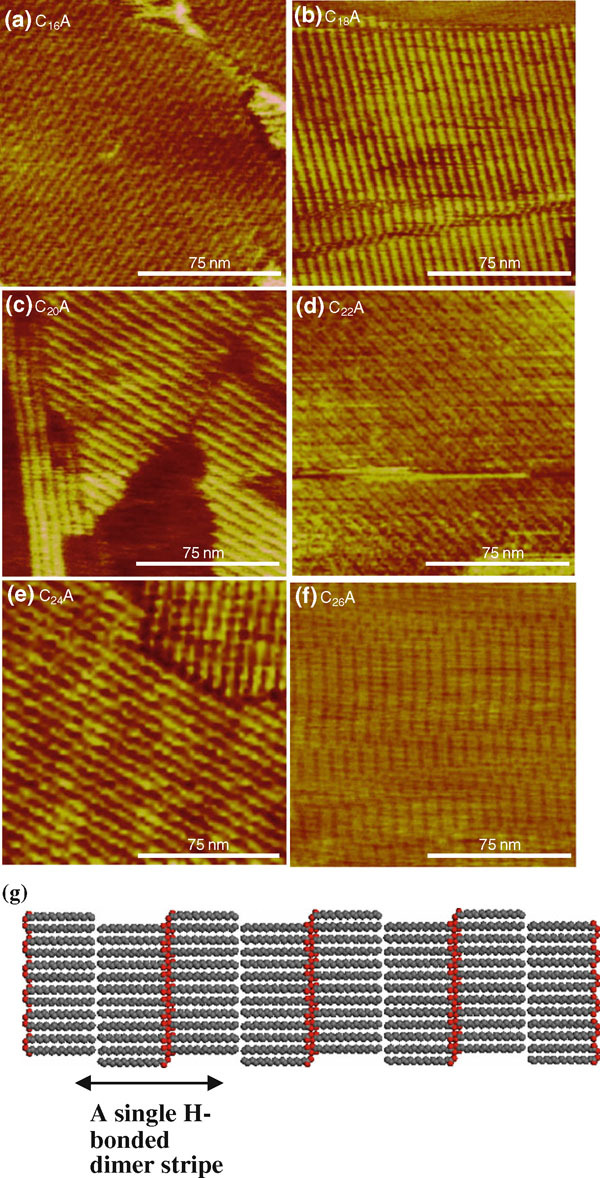
**a–f AFM height images of carboxylic acid monolayers spin coated from alcoholic solvents**. The *z* range is 2 nm for **a**–**c** and 3 nm for **e**–**f**. **g** Molecular packing in 2-D stripe nanopattern of carboxylic acid monolayer on HOPG. The structure is based on C_18_A B-form crystal viewed along the *a* axis. Monoclinic *P2*_*1*_*/a* crystal structure with *a* = 5.591 Å, *b* = 7.704 Å, *c* = 43.990 Å, and *β* = 94.6°.

The spin-coated samples of long-chain *n*-carboxylic acids including hexadecanoic acid (C_16_A), octadecanoic acid (C_18_A), eicosanoic acid (C_20_A), docosanoic acid (C_22_A), tetracosanoic acid (C_24_A), and hexacosanoic acid (C_26_A) were imaged by AFM. When the carboxylic acids were spin coated on HOPG from alcoholic solvents including methanol, ethanol, *iso*-propanol, and *sec*-butanol, only epitaxial stripe nanopatterns were formed (Figure 1). The periodicity of the nanopatterns is 4.5 nm for C_16_A, 5.1 nm for C_18_A, 5.6 nm for C_20_A, 6.1 nm for C_22_A, 6.6 nm for C_24_A, and 7.0 nm for C_26_A. The periodicity is slightly larger than 2 × molecular chain length. The molecular chain length of saturated carboxylic acids on HOPG can be calculated by the following formula: $\left(\frac{1}{2}\text{number}\;\text{of}\;\text{C}\;\text{atoms}\;\text{per}\;\text{chain}+\frac{1}{2}\text{number}\;\text{of}\;\text{O}\;\text{atoms}\;\text{per}\;\text{carboxyl}\;\text{group}\right)\times 0.246\;\text{nm}$. The stripe thickness, 0.3 ± 0.1 nm, is consistent with the coplanar packing model in which the carbon skeleton plane of the carboxylic acid molecule lies parallel to the HOPG basal plane. The orthogonal stripe domains displayed the threefold symmetry of the graphite lattice. It is concluded that the carboxylic acids adopt the persistent epitaxial arrangement on HOPG [4,7,46-49] during spin coating, whose packing structure is illustrated by Figure 1g.

**Figure 2:**
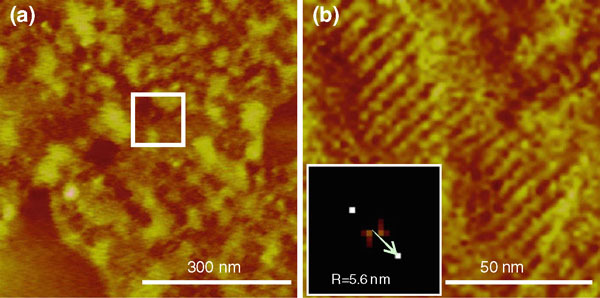
**AFM height images of carboxylic acid spin coated from *m*-cresol (**a**) C_20_A**. **b** Selected area in (**a**). The periodicity was determined by the corresponding 2-D FFT images. The *z* range is 5 nm for both images.

When *m*-cresol was used as the solvent, largely amorphous carboxylic acid films were formed (Figure 2). A closer examination of the AFM images showed ordered domains of C_20_A molecules interspersed in the amorphous film. Clearly, *m*-cresol does not favor carboxylic acid self-assembly either because it is a poor recrystallization solvent for carboxylic acids or because it competes for the adsorption sites on HOPG due to its aromatic group.

**Figure 3:**
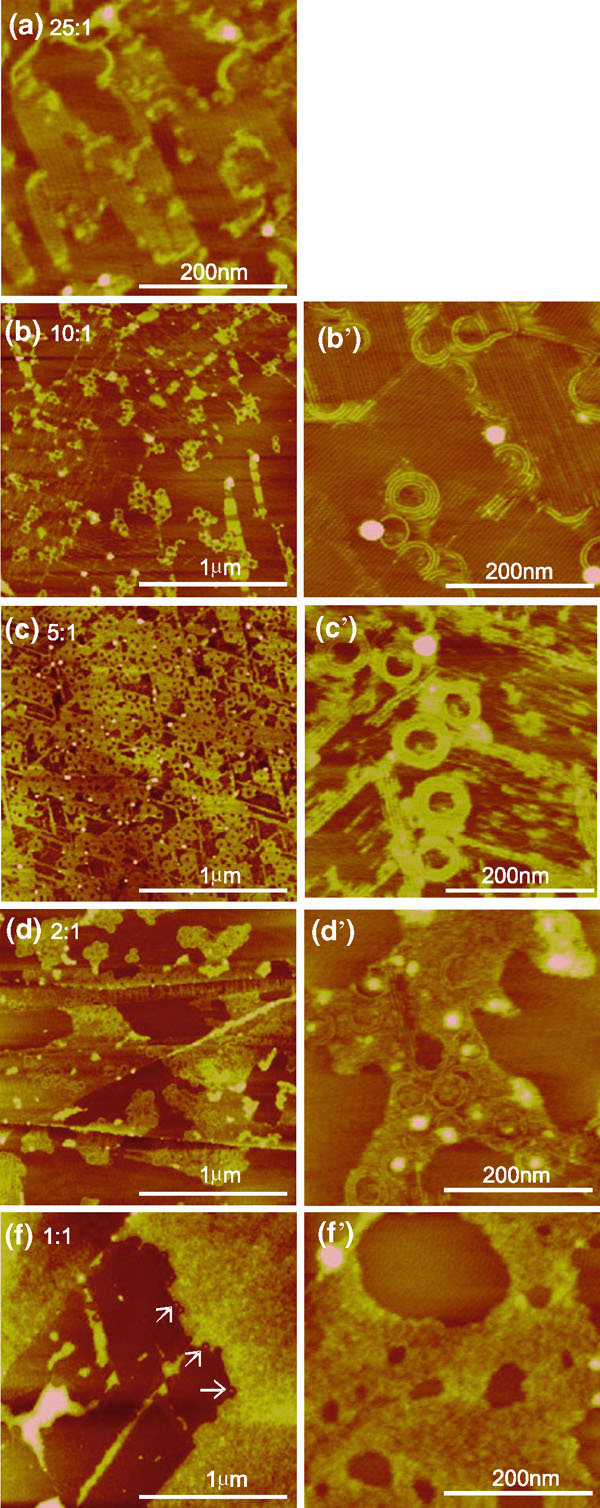
**AFM height images of C_20_A film structures spin coated from methanol and *m*-cresol with different methanol to *m*-cresol volume ratios**. The image on the right is an image with higher resolution than the one to the left. The *z* range is 5 nm for **a**–**e** and 4 nm for **b**'–**e**'.

When *m*-cresol was gradually added to methanol, we obtained new nanostructures in the spin-coated films. Figure 3 shows the typical C_20_A film structures at different methanol to *m*-cresol volume ratios: 25, 10, 5, 2, and 1, respectively. With increasing *m*-cresol content, the film structure changed from highly ordered stripe nanopatterns associated with methanol to circular nanostructures and to disordered phase associated with *m*-cresol. The film coverage increased with increasing *m*-cresol amount. With trace amount of *m*-cresol, the stripe phase was modified by the presence of isolated curved stripes, or partial spirals, that were located either at the edge or on top of the stripe nanopattern (Figure 3a). These spirals mark the locations of partitioned *m*-cresol-rich phase upon solvent evaporation. The curved feature became more prominent with increasing *m*-cresol amount (Figure 3b, b'). The circular stripes are on top of the straight ones. Increasing coverage of the circular feature was obtained with increasing *m*-cresol content (Figure 3c, c'). The circles are uniform in size with an average outer diameter of ~70 nm. In addition to the circles, a straight fiber-like feature is present whose orientation is in registry with HOPG. Each fiber consists of bundles of stripes with height of 0.8 ± 0.1 nm. The straight fiber structure resembles ribbons preceding droplet formation upon reaching the Rayleigh instability limit during dewetting [50,51]. As the ratio decreases to 2, the film became disordered with traces of circular lines (Figure 3d, d'). More *m*-cresol resulted in thicker amorphous films (>1 nm) (Figure 3e, e'). At the edge of the amorphous film, curves were observed as pointed by the arrows in Figure 3e.

**Figure 4:**
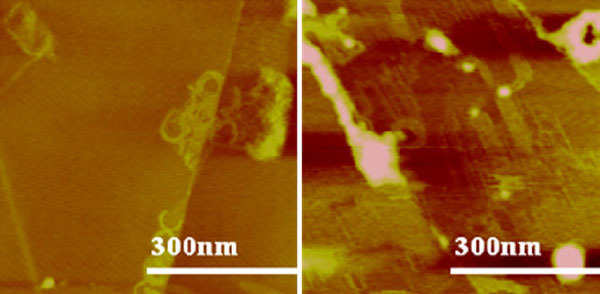
**AFM height images of C_18_A (*left*) and C_22_A (*right*) film structures spin coated from methanol and *m*-cresol mixed solvent (methanol: *m*-cresol = 10)**. The *z* range is 5 nm for both images.

**Figure 5:**
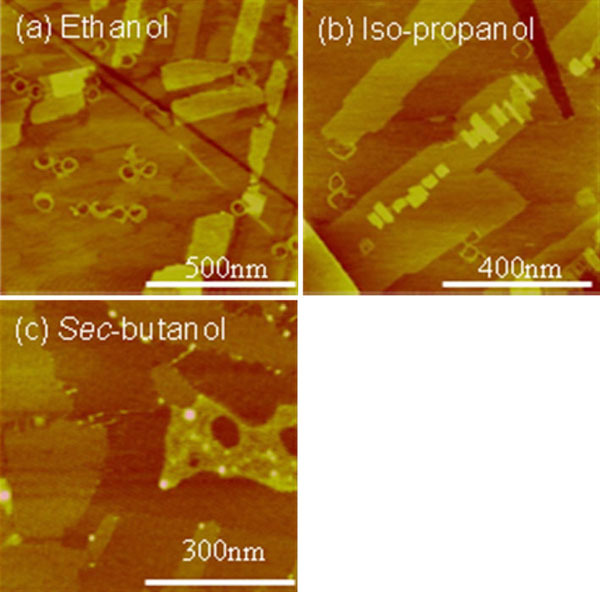
**AFM height images of C_20_A film structures spin coated from ethanol (**a**), *iso*-propanol (**b**), and *sec*-butanol (**c**) with ~10 vol% *m*-cresol**. The *z* range is 3 nm for (**a**) and (**b**) and 5 nm for (**c**).

The circular nanopattern was observed on C_18_A and C_22_A (Figure 4) but not on longer chains. Less-defined spirals were formed when ethanol, *iso*-propanol, or *sec*-butanol instead of methanol was used as the primary solvent (Figure 5). The boundary of the spiral became less circular and more orthogonal. This is a result of two completing templates—the droplet edge versus HOPG basal plane. Less volatile solvents favor epitaxial interaction between the alkyl chain and HOPG lattice.

**Figure 6:**
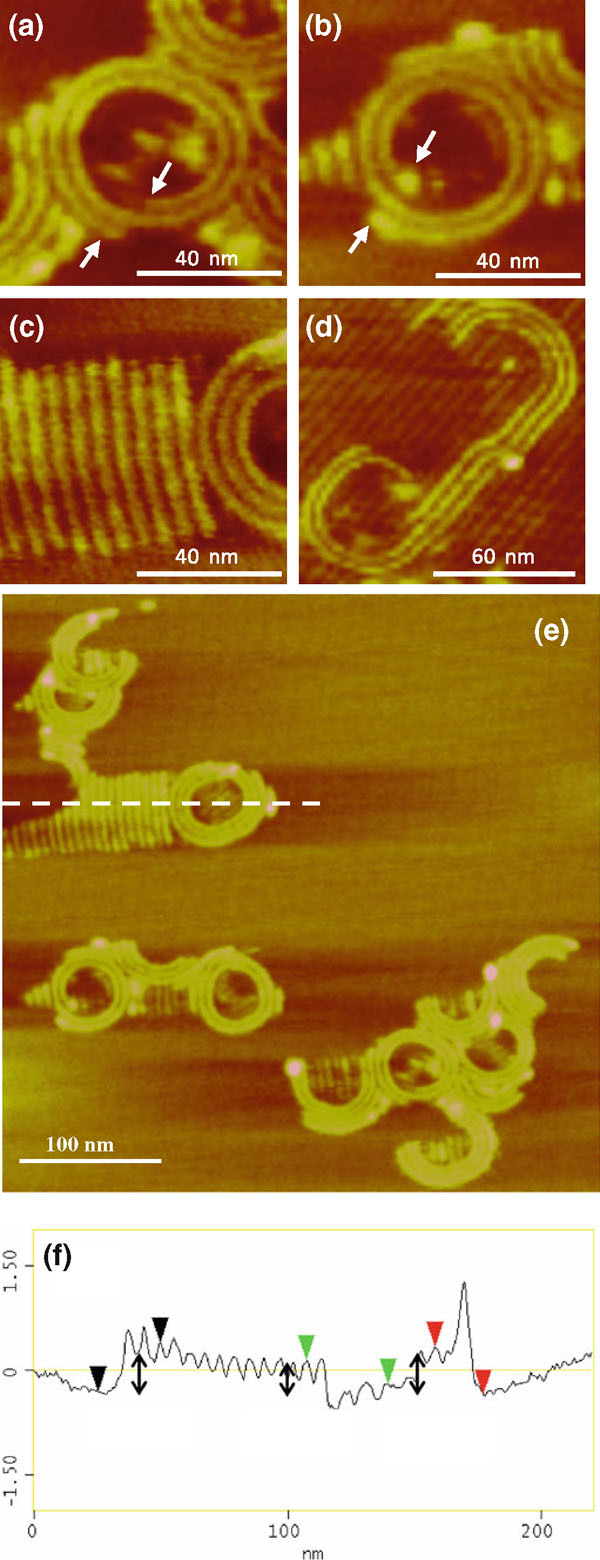
**AFM height images of C_20_A spirals (a–e)**. The *z* range is 4 nm. **f** Sectional height analysis of the stripe height along the *dashed line*.

AFM images at higher resolution using methanol to *m*-cresol ratio of 10 reveal molecular packing structure in the circular nanopattern. Figure 6 provides examples of spirals in inward clockwise (Figure 6a) and counterclockwise rotations (Figure 6b). The arrows mark the beginning and end of each spiral. We found roughly equal numbers of clockwise and counterclockwise spirals. Self-assembled spirals usually involve chiral molecules. Amphiphilic molecules with chiral centers are capable of self-assembly into spirals in Langmuir monolayers. The direction of the spirals depends on the chirality of the amphiphiles. In one study [52], intermolecular H-bonds caused the neighboring aromatic headgroups to tilt and resulted in spiral formation from achiral amphiphilic molecules in Langmuir monolayers. Here, the chirality of the spirals is dictated by the direction of unidirectional solvent evaporation.

Figure 6c–e shows multiple C_20_A spirals, partial spirals, and coexisting straight stripes. The spirals of C_18_A, C_20_A, and C_22_A display a center-to-center distance of 5.1, 5.6, and 6.2 nm, respectively, which indicates that the spiral is made of the same head-to-head dimer arrangement as in the epitaxial stripes on HOPG. The sectional height analysis indicates that the spirals have a uniform height of 0.8 ± 0.1 nm. The straight stripes outside the spiral have the same height as the spirals while those inside tend to have a lower height of 0.2–0.4 nm. The lower height value suggests that the structure is templated only by HOPG in which the carboxylic acid carbon plane faces HOPG [4,53]. The higher height value is consistent with crystalline structure that is not templated by HOPG.

**Figure 7:**
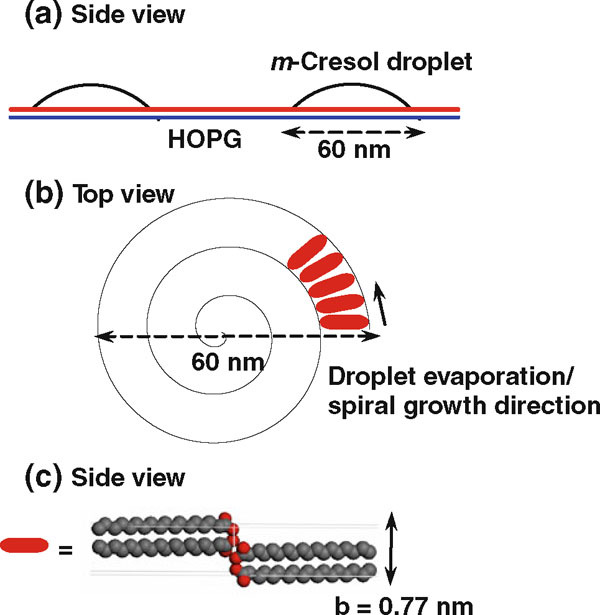
**Schematic mechanism of spiral formation**. **a ***m*-Cresol droplets as templates for the nanospiral pattern. **b** The counterclockwise inward rotating spiral is made of self-assembled carboxylic acid dimers along the evaporating liquid/solid/vapor contact line. **c** Molecular orientation in the spiral on HOPG as represented by the unit cell structure of the B-form C_18_A crystal structure (viewed along the *a* axis). The height of the spiral is close to the unit cell dimension along the *b* axis.

**Figure 8:**
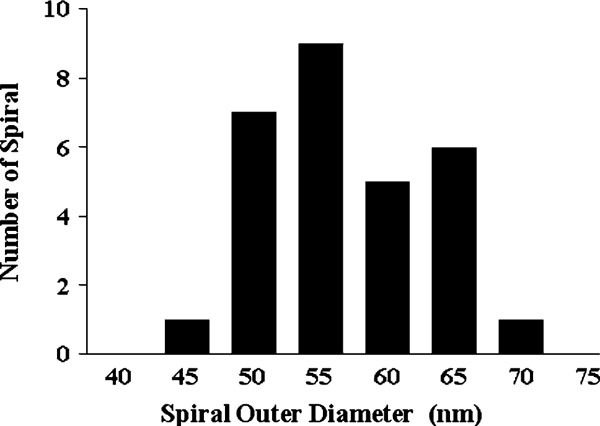
**The size histogram of C_20_A nanospirals (population ~ 30)**.

The spiral nanopattern with a bilayer periodicity suggests that it is templated by precipitation crystallization of carboxylic acids along the receding solid/liquid/vapor interface of an evaporating droplet (Figure 7). In the case of volatile fluid wetting the HOPG substrate, after the outward flow to produce a smooth film, the last stage of spin coating is dominated by solvent evaporation [54,55]. The film thickness is a function of spin speed *f*, initial viscosity *ν*_0_, and evaporation rate *e*: $h\propto f^{-2/3}\nu_{0}^{1/3}e^{1/3}$[54]. In our case, the high spin speed combined with low solution concentration resulted in ultrathin films. When pure solvents were used, the AFM images pointed to uniform thinning of the wetting film until the complete removal of the solvent. The substrate was covered by a uniform carboxylic acid film either in an ordered state from alcoholic solvents or disordered state from *m*-cresol. When the mixed solvent was used, dewetting occurred. Dewetting is believed to start from holes followed by interconnected cellular rims and the breakup of the rims into droplets [51]. Since methanol has higher equilibrium vapor pressure (= 128 mmHg) than *m*-cresol (<1 mmHg) at 25°C, methanol evaporates much faster to yield the stripe layer on HOPG. The remaining *m*-cresol breaks up into small droplets and evaporates at a slower rate enabling molecular self-assembly to proceed. The increase of spiral coverage with increasing *m*-cresol content is consistent with the spiral feature being associated with *m*-cresol. Our results point to the formation of very small and fairly uniform *m*-cresol-rich droplets in the range of 60–70 nm in outermost diameter (Figure 8). The uniform size of the spirals points to a critical film thickness below which the film breaks up into droplets. A rough estimate based on the size of the nanospirals gives a critical film rupture thickness of 4.3 ± 0.3 nm (the contact angle of saturated C_20_A *m*-cresol droplets on HOPG covered by C_20_A nanostripes is 15°).

The drying of solution droplets is described by the coffee-stain mechanism [51,56-59]. The higher evaporation rate at the pinned sessile convex droplet contact edge causes convective capillary flow and precipitation of solute at the edge. The capillary flow goes from the bulk solution to the edge of the droplet in order to maintain the spherical shape to counter evaporative losses [57]. The flow results in solute accumulation at the pinned contact edge as a solid ring. Pinning of the contact line is a "self-pinning" process, which means that the accumulation of the solute at the contact line perpetuates the pinning of the contact line [58]. Multiple rings can result from the solute deposit. An incomplete transfer of solute results in material left inside the ring. Our results show the sequence of this solute deposition for the first time at the molecular scale. The results show that the pinned contact line moves unidirectionally by either a clockwise or counterclockwise inward rotating motion. The process starts with one precipitating H-bonded carboxyl dimer (some spirals have a thicker starting point indicating that sometimes evaporation may start from a cluster of dimers), grows by a crystallization process along a direction normal to the carbon chain and parallel the triple contact line, and terminates with the depletion of either the solute (partial spiral) or solvent (excess deposit of solute as dots inside the spiral).

The length of the spirals provides a measure of droplet concentration at the beginning of droplet evaporation. For example, the total contour length of the spiral in Figure 6b is 272 nm, which corresponds to a total spiral volume of 1.22 × 10^3^ nm^3^ assuming width and height of 5.6 and 0.8 nm, respectively. The B-form C_20_A unit cell size is 1.97 nm^3^ with 4 molecules per unit cell (*a* = 0.549 nm, *b* = 0.740 nm, *c* = 4.855 nm, and *β* = 90°) [60]. Therefore, the total number of molecules in this spiral is 2.48 × 10^3^. Given an outer diameter of the spiral of 56.5 nm, the droplet volume is 4.7 × 10^-21^ L (using 15° contact angle). The C_20_A concentration in the droplet is therefore 0.88 M, a supersaturation of ~ 60 (the C_20_A solubility in *m*-cresol is determined to be ~0.015 M at the room temperature).

The molecular packing structure in the spiral is visualized based on the most stable B-form carboxylic acid crystal structure (C_18_A is used here) [61]. The B-form *n*-carboxylic acid crystal is described as tablet-shaped plate terminated by (001) and (110) faces with interplanar angle of 75° [61-64]. The spiral width direction corresponds to the [001] direction with an interplanar spacing same as 2 × chain length. A likely orientation of the spiral face parallel to HOPG is the (110) face whose interplanar spacing is 0.452 nm. The spiral thickness as determined by AFM is larger, which may mean that the crystalline plane of the spiral face is tilted toward the *b* axis as indicated by the scheme in Figure 7c.

## Conclusions

The unique combination of the binary solvent system and the self-assembling tendency of the carboxylic acids at the interface allow the droplet evaporation process to be captured at the molecular scale. The solid/liquid/vapor interface of *m*-cresol solution droplets serve as templates for the carboxylic acid molecules to self-assemble, which in turn allows the visualization of solution droplet evaporation one molecule at a time. The AFM images show that the pinned contact line moves unidirectionally by either a clockwise or counterclockwise inward rotating motion. The droplet evaporation contributes a new method for the nanospiral formation.
